# Predicting the outcome of death by CALL Score in COVID-19 patients

**DOI:** 10.1590/1806-9282.20230688

**Published:** 2024-03-04

**Authors:** Marcus Villander Barros de Oliveira Sá, Clarice Neuenschwander Lins de Morais, Rafaela Silva Guimarães Gonçalves, Camila Sarteschi, Luydson Richardson Silva Vasconcelos

**Affiliations:** 1 Royal Portuguese Charitable Hospital, Royal Medical Clinic – Recife (PE), Brazil.; 2 Aggeu Magalhães Institute, Oswaldo Cruz Foundation, Department of Parasitology and Immunology – Recife (PE), Brazil.; 3 Universidade Federal de Pernambuco, Clinical Hospital – Recife (PE), Brazil.

**Keywords:** COVID-19, ROC analysis, Death, Models, statistical

## Abstract

**OBJECTIVE:**

The aim of this study was to assess the performance of the CALL Score tool in predicting the death outcome in COVID-19 patients.

**METHODS:**

A total of 897 patients were analyzed. Univariate and multivariate logistic regression analyses were conducted to determine the association between characteristics of the CALL Score and the occurrence of death. The relationship between CALL Score risk classification and the occurrence of death was also examined. Receiver operating characteristic curve analysis was performed to identify optimal cutoff points for the CALL Score and the outcome.

**RESULTS:**

The study revealed that age>60 years, DHL>500, and lymphocyte count ≤1000 emerged as independent predictors of death. Higher risk classifications of the CALL Score were associated with an increased likelihood of death. The optimal CALL Score cutoff point for predicting the death outcome was 9.5 (≥9.5), with a sensitivity of 70.4%, specificity of 80.3%, and accuracy of 80%.

**CONCLUSION:**

The CALL Score showed promising discriminatory ability for death outcomes in COVID-19 patients. Age, DHL level, and lymphocyte count were identified as independent predictors. Further validation and external evaluation are necessary to establish the robustness and generalizability of the CALL Score in diverse clinical settings.

## INTRODUCTION

In December 2019, the World Health Organization (WHO) was notified of an outbreak of an unidentified etiology of atypical pneumonia in Wuhan, Hubei Province, China. Subsequently, on January 30, 2020, the WHO designated the epidemic caused by the severe acute respiratory syndrome coronavirus-2 (SARS-CoV-2) as a public health emergency of international concern^1^. Throughout the course of the coronavirus pandemic, several risk-scoring systems have been developed with the objective of predicting the likelihood of death. These scoring systems aim to identify patients who are more likely to require intensive care support and are at higher risk of mortality^2-9^. One such risk prediction tool is the CALL Score, devised by Ji et al., to assess the need for intensive care in patients with COVID-19 pneumonia^9^. Their study evaluated four variables in a cohort of 208 patients: age >60 years, lymphocyte count ≤1000, elevated lactate dehydrogenase (LDH) levels, and the presence of comorbidities. Comorbidities included hypertension, diabetes, cardiovascular disease, liver disease, asthma, chronic pulmonary disease, HIV infections, and malignancy within the past 6 months. The combination of these CALL Score variables demonstrated reliable predictive capability for disease progression. The ability to predict death is unknown. Concerns have been raised regarding potential overestimation of the score, as it was developed using a small population and relies on only four variables^10^. To ensure transparency and generalizability, further investigations should be conducted involving diverse populations and larger sample sizes.

Despite the WHO’s announcement declaring the end of the COVID-19 pandemic in 2023, global outbreaks persist, and the emergence of novel pandemics remains an unpredictable threat. Therefore, it is paramount to sustain research efforts aimed at deepening our understanding of the disease and devising effective strategies for prevention, management, and ultimate eradication. Identification of risk predictors that accurately estimate the probability of death would assist clinicians in determining the appropriate allocation of resources, ensuring optimal care for patients, and facilitating early transfer to tertiary care facilities when warranted.

## METHODS

A retrospective study was conducted at Real Hospital Português de Beneficência in Pernambuco, Brazil, a private institution catering to patients with various health coverage types, including public (Unified Health System), private, and philanthropic. The study aimed to evaluate the applicability of the CALL Score in predicting death in COVID-19 patients. The study period encompassed patients hospitalized between February 2020 and April 2021. Medical records of COVID-19 patients with confirmed RT-PCR results, who were admitted to the ward and subsequently discharged (either to home or deceased), were reviewed.

Exclusion criteria comprised patients under 18 years of age, pregnant women, patients initially admitted to the ICU, and those with incomplete medical records. Each patient’s medical record was assessed to determine the presence or absence of the four risk factors defined by Ji et al., with appropriate scoring according to their definitions^9^. Comorbidity was defined as the presence of at least one of the following conditions: hypertension, diabetes, cardiovascular disease, liver disease, asthma, chronic pulmonary disease, HIV infections, and malignancy within the past 6 months.

The primary outcome of interest was the occurrence or death. Descriptive statistics, including relative frequencies (percentages) and absolute frequencies (N), were calculated to characterize the study sample. For quantitative variables, means, medians, standard deviations, and minimum and maximum values were employed to summarize the data’s variability. To investigate the associations between variables and outcomes, Pearson’s chi-square test was utilized. The receiver operating characteristic (ROC) curve methodology was applied to determine the CALL Score cutoff points for death outcome. Statistical analysis was conducted using the Statistical Package for the Social Sciences software, version 21.0 (IBM, Armonk, NY).

The research protocol was approved by the Research Ethics Committee of Real Hospital Português de Beneficência in Pernambuco under the registration number CAAE: 38769720.3.0000.9030. The study was conducted in adherence to the principles outlined in the Declaration of Helsinki.

## RESULTS

A total of 1,572 hospitalized patients were admitted between February 2020 and April 2021. Among them, 675 patients were excluded from the analysis as they did not meet the inclusion criteria. The final cohort for analysis consisted of 897 patients who tested positive for COVID-19 confirmed by reverse transcription polymerase chain reaction (RT-PCR) in nasal swab samples and were admitted to the ward. During the course of the study, 27 deaths were recorded among the included patients, while the remaining 870 patients were discharged.

Patient characteristics are described in Table 1.

**Table 1.** Baseline characteristics.

Total number of patients	n=897
Male sex, n (%)	517 (57.6)
Mean age (SD)	51.8 (15.4)
Age, range (years)	20–105
CALL Score, n (%)	
A (4–6)	351 (39.1)
B (7–9)	356 (39.7)
C (10–13)	190 (21.2)
Lymphocytes <1000, n (%)	267 (29.8)
LDH, n (%)	
≤250	529 (59.0)
251–500	348 (38.8)
>500	20 (2.2)
Comorbidities, n (%)	
Chronic heart disease	76 (8.5)
Chronic pulmonary disease	70 (7.8)
Chronic kidney disease	19 (2.1)
Liver disease	4 (0.4)
Chronic neurological disorder	26 (2.9)
Malignant neoplasm	17 (1.9)
Diabetes	194 (21.6)
Rheumatologic disorder	13 (1.4)
Dementia	15 (1.7)
Hypertension	346 (38.6)
ICU admission, n (%)	160 (17.8)
ICU mortality, n (%)	26 (16.3)
Total in-hospital mortality, n (%)	27 (3.0)
Discharged alive – home, n (%)	870 (97.0)

Univariate and multivariate logistic regression analyses were conducted to determine the association between the four clinical and laboratory characteristics of the CALL Score and the outcome “Occurrence of Death” (Table 2). Our findings revealed that age>60 years, presence of comorbidity, DHL>500, and lymphocyte count ≤1000 are independent predictive factors for the occurrence of death in COVID-19 patients admitted to the ward.

**Table 2.** Univariate and multivariate logistic regression analyses of the clinical and laboratory characteristics of the CALL Score and their relationship with the death outcome.

Variable	Death			
	Univariate analysis		Multivariate logistic regression analysis	
	OR (95%CI)	p-value	OR (95%CI)	p-value
Comorbidities*	3.22 (1.74–5.97)	0.008	1.82 (0.66–5.01)	0.244
Age>60 years	4.20 (2.62–8.48)	<0.001	3.05 (1.26–7.41)	0.013
LDH>500 (U/L)	6.28 (3.69–10.70)	<0.001	17.17 (4.97–59.34)	<0.001
Lymphocytes ≤1000 (cell/mm³)	5.93 (3.93–9.03)	<0.001	6.28 (2.55–15.43)	<0.001

* Comorbidities: hypertension, diabetes, cardiovascular disease, liver disease, asthma, chronic pulmonary disease, HIV infections, and malignancy in the past 6 months.

**Figure 1.**
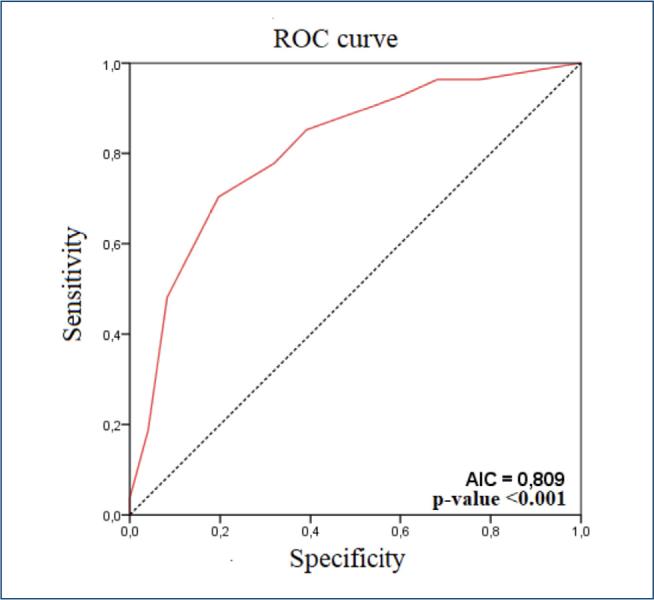
Receiver operating characteristic curve for the death outcome according to the CALL Score.

The CALL Score cutoff point that provided the highest sensitivity and specificity combination for the outcome of death was determined to be 9.5 (≥9.5). This cutoff achieved a sensitivity of 70.4% and a specificity of 80.3%. The positive predictive value was 10%, the negative predictive value was 98.9%, and the accuracy was 80% (Figure 1). The positive likelihood ratio was 3.57, and the negative likelihood ratio was 0.37. The Hosmer-Lemeshow goodness-of-fit test resulted in a chi-square value of 2.9 with a p-value of 0.814, indicating that the CALL score model fits well with the outcome.

## DISCUSSION

To the best of our knowledge, this study represents the first examination of the CALL Score tool within a Brazilian population and also boasts the largest sample size, comprising 897 participants.

The study identified a CALL Score cutoff point of 9.5 for predicting the outcome of death, which yielded an optimal combination of sensitivity (70.4%) and specificity (80.3%). These results suggest that the model exhibits promising discriminatory ability in identifying individuals at risk of mortality while accurately classifying those who survive. The PPV of 10% implies that caution should be exercised when interpreting positive predictions, as only a small proportion of patients predicted as positive by the model experienced the outcome of death. However, the high NPV of 98.9% indicates the model’s effectiveness in ruling out the risk of death for patients predicted as negative. The overall accuracy of 80% demonstrates the model’s reasonable performance in correctly predicting the outcome of death. Nevertheless, further investigation is warranted to validate its robustness and generalizability against the existing models or criteria in the field. The positive likelihood ratio of 3.57 suggests that a positive prediction by the model moderately increases the odds of death. Conversely, the negative likelihood ratio of 0.37 indicates a moderate reduction in the odds of death for patients predicted as negative by the model. These findings contribute valuable insights into the predictive capacity of the model for death outcomes. However, it is imperative to consider the clinical implications, potential limitations, and the need for external validation to establish its reliability and applicability in diverse clinical settings.

A comprehensive review of the existing scientific literature on the CALL Score tool has been conducted. In a study conducted at the University Hospital of Turkey involving 256 patients, the CALL Score did not demonstrate reliability in predicting progression to severe disease or death, exhibiting an area under the ROC curve of only 0.59 (95%CI 0.50–0.66)^11^. In Wales, where the CALL Score was applied to 169 patients across three levels, it failed to serve as a reliable predictor of death, with an area under the ROC curve of 0.500 (95%CI 0.411–0.589)^12^. The limited sample size of these studies may have influenced the performance of the tool.

Conversely, in an Italian population of 210 patients, the CALL Score demonstrated effectiveness as a prognostic index for in-hospital mortality^3^.

Validation of the CALL Score in a Pakistani population comprising 252 patients confirmed its reliability as a prognostic indicator for mortality in COVID-19 patients^13^. However, in the multivariate analysis, the presence of comorbidity was not found to be a reliable independent risk factor for disease progression, which contrasts with several studies that have correlated the pre-existing comorbidities with worsened disease and mortality in coronavirus patients^14^. It should be noted that obesity was not considered a comorbidity in the CALL Score tool. This is likely because obesity was only described as a risk factor in the literature after the publication of the tool. Previous studies, including Kamran et al., have also failed to demonstrate the significance of comorbidities as risk factors in multivariate regression analyses with CALL Score variables. The significance of the pre-existing medical conditions in COVID-19 patients is now well recognized. The variables such as age>60 years, DHL>500, and lymphocyte count ≤1000 were consistently identified as reliable independent risk factors for disease progression and death, aligning with the findings from previous studies^1,7,8,14-20^.

It is imperative to acknowledge the inherent limitations of our study, which include its monocentric nature and retrospective design. Moreover, the evaluation of the tool was restricted to patients admitted directly to the ward, warranting caution in its application to outpatients or those admitted directly to the ICU.

## CONCLUSION

This study evaluated the performance of the CALL Score tool in predicting the death outcome in COVID-19 patients. Our findings suggest that the CALL Score demonstrates promising discriminatory ability for death outcomes. Age>60 years, DHL>500, and lymphocyte count ≤1000 emerged as independent predictors of ICU admission and/or death, while the presence of comorbidity did not show a significant correlation with death outcomes. The CALL Score risk classification exhibited a positive correlation with death, with higher risk classifications associated with an increased likelihood of this outcome. Further validation and external evaluation are necessary to establish the robustness and generalizability of the CALL Score in diverse clinical settings. Overall, these findings contribute to the understanding of prognostic tools in COVID-19 and emphasize the need for continued research in this field.
